# Computational studies on the molecular insights of aptamer induced poly(*N*-isopropylacrylamide)-*graft*-graphene oxide for on/off- switchable whole-cell cancer diagnostics

**DOI:** 10.1038/s41598-019-44378-x

**Published:** 2019-05-27

**Authors:** Athika Darumas Putri, Bayu Tri Murti, Suvardhan Kanchi, Myalowenkosi I. Sabela, Krishna Bisetty, Ashutosh Tiwari, Inamuddin, Abdullah M. Asiri

**Affiliations:** 1 Department of Chemistry, Faculty of Applied Science, Durban University of Technology, Durban, 4000 South Africa; 2 Semarang College of Pharmaceutical Sciences, Jl. Letnand Jendral Sarwo Edi Wibowo, Semarang City, 50192 Indonesia; 3 Institute of Advanced Materials, UCS, Teknikringen 4A, Mjärdevi Science Park, SE-58330 Linköping, Sweden; 4 Vinoba Bhave Research Institute, Binda-Dhokri Road, Saidabad, Allahabad, 221508 India; 5 Chemistry Department, Faculty of Science, King Abdulaziz University, Jeddah, 21589 Saudi Arabia; 6 Centre of Excellence for Advanced Materials Research, King Abdulaziz University, Jeddah, 21589 Saudi Arabia

**Keywords:** Environmental impact, Bioanalytical chemistry

## Abstract

This work deals with first-principles and *in silico* studies of graphene oxide-based whole-cell selective aptamers for cancer diagnostics utilising a tunable-surface strategy. Herein, graphene oxide (GO) was constructed as a surface-based model with poly(*N*-isopropylacrylamide) (PNIPAM) covalently grafted as an “on/off”-switch in triggering interactions with the cancer-cell protein around its lower critical solution temperature. The atomic building blocks of the aptamer and the PNIPAM adsorbed onto the GO was investigated at the density functional theory (DFT) level. The presence of the monomer of PNIPAM stabilised the system’s π-π interaction between GO and its nucleobases as confirmed by higher bandgap energy, satisfying the eigenvalues of the single-point energy observed rather than the nucleobase and the GO complex independently. The unaltered geometrical structures of the surface emphasise the physisorption type interaction between the nucleobase and the GO/NIPAM surface. The docking result for the aptamer and the protein, highlighted the behavior of the PNIPAM-*graft*-GO  is exhibiting globular and extended conformations, further supported by molecular dynamics (MD) simulations. These studies enabled a better understanding of the thermal responsive behavior of the polymer-enhanced GO complex for whole-cell protein interactions through computational methods.

## Introduction

Carbon-based structures are unique materials widely used in various fields due to excellent thermal, mechanical, and electrical properties. Moreover, the interactions with biomolecules harness the benefits in numerous applications, particularly in the field of biosensors, medical diagnostics and drug delivery systems^1–5^. Its applications regarding graphene and graphene oxide are expected to be less toxic at low concentrations. Hence, they are readily applicable to biomedical functionalities. Graphene oxide (GO) is an exfoliated single-layer graphene sheet with oxygen functional groups distributed along its surface. It has gained widespread recognition due to its excellent conductivity and feasibility for microfabrication, large surface area, high optical transparency, and surface biocompatibility in biomedical applications^6–9^. These properties are attributed to its unique contents which consist of sp^2^ hybridised carbon and sp^3^ hybridised carbon and oxygen functional groups surrounding the surface. GO, is widely associated with DNA through its non-covalent interactions, thereby generating a complex intercalated structure^10,11^.

A synthetic oligonucleotide (either RNA or DNA) populated nucleobases, from a systematic evolution of ligands by exponential enrichment (SELEX), is widely known to be a highly selective-targeting agent towards any molecules in sensing applications ranging from small molecule protein to whole cell body^12^. Nucleobases are the main parts of the aptamer building-unit which reflects the core chemical behavior of the complex biomolecules. Since the simple piece of a double-stranded or single-stranded DNA of the aptamer plays a role in biological processes, it is essential to understand the interactions between the nucleobases with the nanomaterials. There have been several theoretical studies for the nucleobase adsorption to discover the binding characters and the relative binding strength of the four DNA bases towards graphene and carbon nanotubes. For instance, the previous DFT studies using the local density approximation (LDA) demonstrated that the relative binding energies presented by nucleobases on the hexagonal carbon nanosurface were in the order of G > A > T > C, and they were comparable with calorimetric titration results^11,13–16^. Indeed, these studies allow for simplifying the intricacies of the DNA complex adsorbed onto the carbon nanostructures to gain an in-depth understanding of the binding mechanisms between the biomolecules and the carbon nanostructures.

A polymer is a chain of a repeated single molecule (or monomer) connected through chemical or physical bonds. Due to their physical-chemistry properties, polymers are being evaluated for many biomedical applications as a supporting system/device to gain more effective and accurate results^17,18^. The well-known smart polymer, poly(*N*-isopropylacrylamide) (PNIPAM), has gained tremendous interest in numerous applications due to its low critical solution temperature (LCST), around that of the human body temperature of 32 °C. This functional PNIPAM polymer retains its solvated coiled structure in the water below LCST, while it undergoes adesolvated globular state at temperatures above the LCST. This feature also led to its widespread applications particularly in diagnostics, drug delivery, regenerative medicine and tissue engineering^17,19–26^. For instance, the thermal-responsive characteristic of PNIPAM was utilised in triggering the biorecognition interaction with its target^27,28^. Indeed, this functional polymer has been ubiquitously used in the development of various nanomedical biosensors^29,30^. Regarding cancer detection, PNIPAM is commonly used to study the interaction between biorecognition and the target at different temperatures or pH. Accordingly, a sensing technique was developed by Kim and co-workers describing PNIPAM-*co*-acrylic acid (PNIPAM-AAc) microgel in a microlens array for tunable protein detection^31,32^. These systems benefited from the temperature and pH functions where the water content of the microgel reflected the lensing ability due to changes in the refractive index of the target protein. Jung and co-workers, on the other hand, proposed PNIPAM-based microfluidics to detect the protein adsorption on the droplet surface^33^. The use of *b*-poly(4-cyanobiphenyl-4′-oxyundecylacrylate) and the PNIPAM (5CB_PNIPAM_) associated with sodium dodecyl sulfate (SDS) in microfluidic components have demonstrated the changes on the radial-to-bipolar (R-B) orientation profile after exposure at an absolute temperature and pH corresponding to the protein adsorption. More recently, Shiddiky and co-workers have developed a PNIPAM based immunosensors that induces a reversible surface for the detection of cancer protein in human serum^34,35^.

The experimental analysis of PNIPAM has been successfully demonstrated and highlighted the role of the PNIPAM monomer in the adsorption of the nucleobase onto the GO, enabling a better understanding of the dynamic behavior of switchable interactive-surfaces of DNA-protein that is triggered by the PNIPAM-grafted GO^31,32^. For this purpose, the modelling of nanomaterials integrated into biosensing systems are essential to enhance the interaction of the performance between biorecognition and its target under a particular environment.

To the best of our knowledge, there is no report available in the literature on the calculation of the electronic properties contributing to the nanomaterials integrated “on/off” switchable aptasensor system. Herein, we report a density functional theory (DFT) study on the structure contribution of the monomer of PNIPAM grafted onto the GO surface and its effective adsorption of the nucleobases. This investigation involved the interaction of the PNIPAM-grafted GO surface with an immobilised aptamer. The PNIPAM molecule was represented by a single monomer (NIPAM), functionalized onto the GO flake as GO/NIPAM complex. Whereas, the aptamer was depicted by a single nucleobase adsorbed onto the surface of the GO/NIPAM complex. Nucleobases are the elementary building blocks of the nucleic acids that enables it to reflect the general chemical properties of intricate biomolecules^36^. Generally, the obtained computational data would comprehend the biomolecular interactions with organic molecules on the nanomaterial surface, essential in the construction of an effective biosensor with high specificity^13,37^. This study enabled biosensor research to be undertaken in the field of biomedical applications, especially in terms of the binding strength and electroconductivity in the presence of an insulator monomer, NIPAM^11,13,37–40^.

Additionally, molecular docking studies were performed with an aptamer for a better understanding of the interaction between oligonucleotides and the target cells. Further, the simulations of the protein binding with the DNA were carried out using the PNIPAM and GO nanostructure.

To the best of our knowledge, this is the first theoretical study to assess the PNIPAM effects on the adsorption of nucleobases as well as its biomolecular interaction with GO surface. The effect of the PNIPAM on the nanomaterial surface experimentally does not contribute much to the full electronic feature of the system due to the insulating nature of the polymer. However, our results indicate that the bandgap energy distribution before and after the functionalisation of the PNIPAM monomer remains unchanged.

## Materials and Methods

### Density functional theory

The GO model was constructed based on the previous models containing one epoxide and three hydroxyl groups on the basal plane and a carboxyl group at the border^11,41^. The PNIPAM monomer was modelled as a single molecule and subsequently grafted as a functionalised molecule on the GO surface through the carboxyl conjunction. The nucleobases were individually sketched and placed in parallel above the functional groups of either GO or GO/NIPAM surfaces. The initial distance between the nucleobase molecule and the functional groups was set to 2.5 Å before weak bonding interaction.

The initial geometries of each structure and their complex systems were calculated using the DMol3 package along with the calculation of the electronic properties employing the generalised gradient approximation (GGA) with the Perdew-Burke-Ernzerhof (PBE) functional^42^. Local density approximation (LDA) does not fully account for the dispersion forces among molecules and may contribute to the negligible overlap between the electron densities and its overestimated interaction energies. This is overcome by the application of GGA since it describes better electronic interactions between the two subsystems and corrects the overestimated interaction energy produced by LDA^43–45^. On the other hand, the PBE functional was employed to explain a weak interaction ruled by hydrogen bonding and van der Waals interactions^46–48^. The double numerical quality basis set with the polarised orbital (DNP) was applied to describe all electron Kohn-Sham functions, where the 2s and 2p orbitals are employed for the carbon atoms. Further, the DNP function set is analogous to the 6-31G** basis set, and since it is based on the atomic orbitals, the results are expected better than using Gaussian basis. Self-consistent field (SCF) tolerance was set to 10^−6^ eV/atom with 1,000 iterations, while the Hartree energy tolerance, force and the maximum displacement energy were set to 10^−5^ eV, 2.0 × 10^−3^ eV/Å, and 5.0 × 10^−3^ Å, respectively. To accurately define the van der Waals and hydrogen bonding correlations, DFT-dispersion (DFT-D) option was employed using the Tkatchenko and Scheffler (TS) functional, in which the nucleobase has adhered onto the GO surface^49^. For this purpose, the hybrid functional was excluded since it was not computationally efficient for a single system calculation. The adsorption energy (*E*_*ads*_) was calculated using:

$${E}_{ads}={E}_{GO(/GO-NP)}+{E}_{nucleobase}-{E}_{-complex}$$

where the *E*_*GO(/GO−NP)*_, *E*_*nucleobase*_, *E*_*−complex*_ belong to the total energies of either GO or GO/NIPAM surface; single molecule of nucleobase (adenine (A), guanine (G), thymine (T), or cytosine (C)); as well as complex formation between the nucleobase and surface, respectively. Herein, the counterpoise correction was not involved during the calculation of the adsorption energy under DMol3, since the DNP numerical basis set was employed to minimise the basis set superposition error^50^. All calculations were treated with the all-electron core employing SCF with the convergence criteria of 10^−6^ a.u. In this correlation, the chemical structure of the *N*-electron system can influence the reactivity profile of the molecule, which further explains its electronegativity is directly proportional to its ability in accepting the electrons.

Therefore, the quantum descriptors for the calculations of the system were defined as follows^50^:

$$\chi =-\,{(\frac{\delta E}{\delta N})}_{v(\overrightarrow{r})}=-\mu $$

While the second derivative of the energy toward the external potential $v(\overrightarrow{r})$ is expressed as hardness *(η*) in:

$$\eta =\frac{1}{2}{(\frac{{\delta }^{2}E}{\delta {N}^{2}})}_{v(\overrightarrow{r})}$$

The equations of each *μ* and *η* are described using the Mulliken principle^51^ in the restricted method, in which, they are correlated with the potential of ionisation *(I)* as well as the electron affinity *(A)*:

$$\mu =-\,\chi =\frac{1}{2}(I+A)$$

and

$$\eta =\frac{1}{2}(I-A)$$

*I* and *A* is equivalent to the −E_HOMO_ and −E_LUMO_, respectively^52^.

The geometry optimisation of a single molecule and each complex system was configured to attain the lowest energy state of the structure. During the optimisation, the position of the atoms could relax to decrease the total energy of the system. The iteration cycle occupied during the process determines the potential gradient at each atomic position to be minimised. The convergence tolerance employed will fit with each iteration, thus yielding the minimum energy between two successive iterations which are less than the convergence tolerance. The electronic properties derived from this calculation are the density of states, highest-occupied molecular orbital (HOMO), lowest-unoccupied molecular orbital (LUMO), electrostatic potential, total energy, and the vibrational spectra.

### Molecular dynamics simulation

The aptamer and protein binding site was firstly determined through molecular docking (see Supporting Information) using the ZRANK module of Discovery Studio. The aptamer sequence was adapted from Wang and coworkers^53^, whereas the whole-cancer cell protein was extracted as a PDB file from the RSCB Protein Data Bank. The resulting binding site was subsequently used to define the binding poses for the MD simulation. The GO complex was constructed as a 2D model consisting of 1271 carbon atoms with the epoxy and hydroxyl groups present on both surfaces, and the carboxyl groups randomly assembled on the edges of the structure (i.e. C_10_O_1_(OH)_1_(COOH)_0.5_). The oxygen functional groups on the GO complex was based on the outcome of a standard oxidation process^54–56^. Two PNIPAM chains with repeating units of 50 monomers each (MW: 5660) were constructed using the optimised monomer structure. The PNIPAM chains were assembled onto the GO surface via carboxylic conjunctions on the middle of the wider edges of GO. The GO surface, PNIPAM chains, Wy5a aptamer and the α6β4 protein were individually optimised before incorporated into a periodic boundary cell (PBC), using the Universal force field (UFF) due to its inherent capacity in calculating many combinations of atoms^57^. Around 50,000 steps of smart minimization were applied to converge the system to a value of 0.001 kcal/mol with a force of 0.5 kcal/mol/Å. The UFF was chosen as a force field for large biomolecules with hydrogen bonding within the biomolecules^58^. Accordingly, the results of the optimised structures were utilised in the MD simulations. The periodic lattice was created with parameters *a* = 15.0 nm, *b* = 12.0 nm, and *c* = 12.0 nm and with the system centrally positioned. A vacuum space of around 15 Å was applied to avoid the artefact effects of non-periodic directions within the PBC box. The optimised Wy5a aptamer from Discovery Studio optimisation was placed approximately 5 Å from the GO surface vertically by considering that the active binding sites were placed away from the GO according to the docking orientation with the α6β4 protein. Using the Amorphous Cell module, the water solvent was packed into the system employing 11,399 water molecules with 122 sodium and 92 chlorine atoms added to neutralise the charges within the system.

To verify the thermal-responsive characteristics of the constructed PNIPAM, the initial simulation of a single PNIPAM chain was firstly carried out. The simulation employed an optimised PNIPAM chain dissolved in water within a PBC system. The MD was performed under NVT ensemble for 10 ns at 298 K and 15 ns at 310.7 K.

The MD of the system was divided into two types of subsequent simulations: *System I*, without α6β4 protein and *System II*, with α6β4 protein. In System I, the protein molecule was not included in PNIPAM-GO-aptamer system. The equilibrium of the System I was performed for 200 ps by keeping the central system fixed while the PNIPAMs and water molecules were allowed to equilibrate. The thermal responsive behavior of the PNIPAM-grafted GO were further investigated by performing MD for 4,800 ps at 298 K and 310.7 K, which correspond to the temperatures below and above the LCST, respectively.

The interaction of a α6β4 protein with Wy5a aptamer on the PNIPAM functionalized-GO surface was set in System II, where the α6β4 protein was placed at a distance of 0.5 nm from the aptamer. Upon protein exposure, the system was kept fixed, and optimisation was subsequently performed using the same parameters as System I. Next, System II simulation was executed for 500 ps with a time step of 1.0 fs, allowing the protein to move freely, while the other elements were constrained. The final results appeared in the trajectories containing the energetics datasheet for the simulation time in every 100 fs. The interaction energies were obtained from a single point energy calculation using the COMPASS force field^59–61^.

All the simulations were performed at constant temperature and volume (NVT). The Nosé-Hoover-Langevin (NHL) thermostat was employed using a coupling time of 1 ps. The Particle-Particle Particle-Mesh Ewald (PPPM) summations were used to evaluate the electrostatic interactions and atom-based cut-off to evaluate the van der Waals interaction. The PPPM was selected since it is suitable to perform the faster calculation for large system^62,63^. Whereas, the atom-based cut-offs has been widely used to compute the long-range non-bond interactions in which the non-bond parameters are calculated based on a selected cut-off value while any interactions beyond the cut-off are not considered^63^.

## Results and Discussion

### Density functional theory

#### Structure and energetics

The DFT calculations were performed to establish the role of the PNIPAM monomer in affecting the electronic properties and the structural energetics of the nucleobases adsorbed onto the GO surface. The systems were built by placing the nucleobases at a distance of approximately 2.5 Å from the oxygen groups containing the modified GO in the π-π stacking positions of the GO surface. The initial configuration of each system of the nucleobase was relaxed, and the minimum energy orientation was selected for the next step in the calculation.

**Figure 1:**
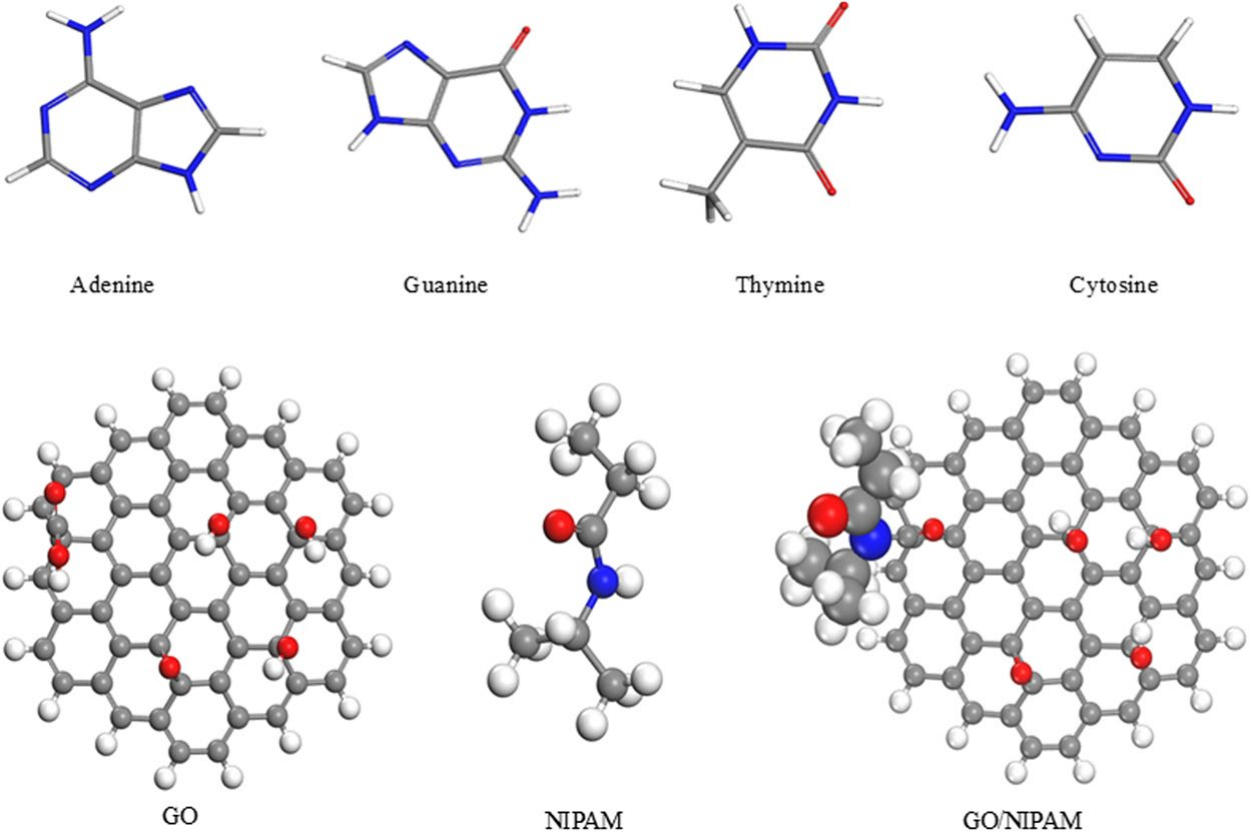
The optimised structure of nucleobases, GO, NIPAM, and GO/NIPAM.

In the case of the structural geometry studies, the nucleobase complexes either with the GO or the GO/NIPAM surfaces and the geometrical changes, atomic hydrogen bonding of nucleobase toward the surfaces and its effect on the adsorption energies are compared. The optimisation of each nucleobase, GO, PNIPAM monomer, and GO/NIPAM structures are depicted in Fig. 1. Since no specific model is assuming GO, the basic structure of GO was shaped in a circular geometry. This kind of structure was selected to hinder the anisotropic effects induced by the size differences of GO in designing a specific direction^41^.

A 1.44 Å bond length was observed for the functionalised C–C bonds of GO and GO/NIPAM upon optimisation for each adjacent carbon atom, in accordance with the previous reports^64–66^. On the other hand, the bonds connecting the NIPAM to the GO complex, C12-C58 and C58-N, changed from 1.548 Å to 1.635 Å and from 1.521 Å to 1.427 Å respectively (Fig. S1). These changes in the bond lengths upon optimisation are necessary to achieve a better conformational profile thus affecting the bond length alteration and the displacement with minor perturbations of the NIPAM in the GO complex. The extended bond length indicates that there is charge transfer from the carbon atoms of GO around the NIPAM molecule to the nitrogen atoms of the NIPAM molecule, thus affecting the weakened C12-C58 after adsorption. The shorter bond length observed for the C58-N atoms suggests a stronger binding with the functionalised GO surface^67^, which supports sp3 hybridisation of the C-N bond^68^.

Similar confirmations were also investigated for the nucleobases adsorption of GO and the GO/NIPAM systems, particularly in the site of the carboxylic functional group’s ends which connects the NIPAM molecule with the GO surface. Attention is given to this to discover the influence of the NIPAM molecule present on the geometrical configuration of the nucleobases in adsorption evidence on the GO surface. The presence of NIPAM exhibit the alterations in term of bond length and bond angle in every interactive system between the nucleobase and the GO surface (Tables S1–S4). The shortened bond length indicates a stronger interaction and higher bond-order of all the bonded electrons between the two atoms.

Obvious differences are found in the bridging angles, especially at the C11-C12-C58 and C58-C12-C28 angles. The changes observed in C11-C12-C58 are 3.771°, 1.283°, 8.891°, and 2.899° for A, C, T and G, respectively (Tables S1–S4). Interestingly, there is a noticeable alteration among other nucleobases regarding the attractiveness that occurred in the corresponding angle of the T system. While three of the nucleobases have increased bond angles, the T system decreased from 109.573° to 100.862°. A strong repulsion between the nitrogen and oxygen atoms of the anions toward the carbon atoms correlates well with an increase of the C58-C12-C28 and a decrease of the C11-C12-C58 angles^69^. These significant changes reflect the most robust interactions exposed in the presence of the T molecule in the GO/NIPAM system.

The initial configuration of each system was relaxed, and the resulting optimised configurations with the minimum energies were selected for the following calculations. The minimum force of the nucleobase-GO/NIPAM and nucleobase-GO structures along with the hydrogen bonds are presented in Figs S2 and S3, respectively. The distance within the nucleobase and GO on both systems are compared before and after nucleobase adsorption. The observed changes in the distances of every nucleobase complex along with their orientations upon the adsorption are presented in Table S5. In this regard, PBE functions using the GGA were useful since it is adequate to describe the weak interactions of van der Waals and hydrogen bond^46^, particularly in the nucleobase system toward the GO molecule^11^.

As presented in Figs S2 and S3, the nucleobase molecules are not in a parallel direction towards the surfaces. It is shown in the optimised complex system that the nitrogen, oxygen, or hydrogen atoms of nucleobase tend to tilt or to head toward the hydroxyl or epoxy functional groups of the surface forming a hydrogen bond, thus attracting the nucleobase closer with the molecular surface. This suggests that those atoms interact mostly with the dangling oxygen groups of the surface during the adsorption. For a molecule in a complex with the GO/NIPAM (Fig. S2), two hydrogen bonds, ${{\rm{N}}}^{\cdots }{\rm{H1}}$ and ${{\rm{H2}}}^{\cdots }{\rm{O1}}$ indicated at 1.775 Å and 2.115 Å respectively, with 1 intramolecular between the hydroxyl groups (${{\rm{H3}}}^{\cdots }{\rm{O2}}$ at 2.032 Å) (Table S5). In contrast to other nucleobases systems, the intermolecular bonds are mostly found in C and T systems. In the C system, the intermolecular bonds; ${{\rm{N}}}^{\cdots }{\rm{H1}}$, ${{\rm{H4}}}^{\cdots }{\rm{O4}}$ and ${{\rm{H3}}}^{\cdots }{\rm{O3}}$ at 2.072, 2.093 and 2.133 Å respectively, along with the intramolecular bond at 2.070 Å (${{\rm{H2}}}^{\cdots }{\rm{O2}}$). In the T system, the bonds observed are within ${{\rm{O1}}}^{\cdots }{\rm{H1}}$, ${{\rm{O1}}}^{\cdots }{\rm{H2}}$, and ${{\rm{H3}}}^{\cdots }{\rm{O2}}$at 1.755, 2.494 and 1.785 Å respectively, as well as the intramolecular bonds ${{\rm{O2}}}^{\cdots }{\rm{H2}}$ at 2.408 Å. Whereas, in the G system, only one intramolecular and intermolecular bonds ${{\rm{O1}}}^{\cdots }{\rm{H2}}$ and ${{\rm{O2}}}^{\cdots }{\rm{H1}}$ at 2.029 Å and 2.180 Å respectively. Similar calculations have been applied to the nucleobases in the GO surface complex. Fig. S3 shows the optimised geometries of the nucleobases-GO complexes along with the hydrogen bonding (dashed blue colour). It is observed that almost all the nucleobase structures have four hydrogen bonds within the GO complex which may suggest more physical interactions occurring within the GO complex rather than those within GO/NIPAM. Based on these results, the interactions between the nucleobase and GO or GO/NIPAM are mostly stabilised by hydrogen bonded interactions.

It is noteworthy that all adsorption energies observed in GO as well as in GO/NIPAM complexes are negative, implying that the adsorption of the nucleobases on both surfaces are in stable configurations and highly exothermic (Fig. S4 and Table S6), corresponding to a more energetically preferable states^70^. It was noted that the functional energetics among the systems, in terms of adsorption energy was favorable. The trend shown in nucleobase-GO/NIPAM complexes is in the order of T > G > C > A. The different adsorption energy is due to the variety of electrostatic interaction among the nucleobase systems. Higher adsorption energy corresponds to the stronger electrostatic attraction between the positive and negative charges of the nucleobase and the oxygen groups of GO, respectively. Whereas, the decrease in adsorption energy is attributed to the repulsive forces of the electrostatic interactions between the delocalised π electrons and oxygen lone pair electrons^36,71^. The variation in the adsorption energies is also due to the number and strength of the hydrogen bonding. Among the nucleobase-GO/NIPAM complexes, T system has the highest adsorption energy as it favors a high number of hydrogen bonds with shorter distances among other nucleobase-GO/NIPAM systems. Analogues with this trend, the T system has the most attractive configuration toward GO/NIPAM surface due to the noticeable changes indicated in the bond angle of the surface geometry.

Similar calculations were performed for the GO complex, with increasing adsorption energy in the order of G > A > T > C, in accordance with the experimental report^72,73^ and previous DFT studies^13,16,71,74^. The adsorption energy of each nucleobase complex seems quite close with each other (around~100 kJ/mol) with G as the highest binding energy. The adsorption energies in the nucleobases-GO systems are influenced by the presence of the hydrogen bonds observed within their complexes corresponding to the similar high numbers of bonds (four hydrogen bonds). Furthermore, the adsorption energies found are relatively higher than those in nucleobases-GO/NIPAM complexes, due to the absence of NIPAM molecule. Thus, the electrostatic forces are more polarised within the nucleobase and oxygen functional groups of GO in the complex. However, the adsorption energies found in GO/NIPAM complexes are within the range of negative eigenvalues, suggesting that the adsorption is energetically favorable in all cases of nucleobases. Finally, from the adsorption energies and the equilibrium distances, the results suggest a moderately strong interaction between the nucleobases and both surfaces, in accordance with physisorption.

#### Electronic properties

The calculated values for the frontier orbitals of the individual elements and the GO/NIPAM complex, nucleobases-GO, and the nucleobases-GO/NIPAM are presented in Table S7. The calculated band gap energies of the GO, NIPAM, GO/NIPAM are 0.631, 5.124, 0.656 eV, respectively, while the band gap energies for the nucleobase molecules of A, T, G, and C are 3.886, 3.820, 3.951, and 3.690 eV respectively.

It is well-known that GGA underestimates the band gap energy calculations, whereas the real band gap energies are expected to be higher^75^. The results suggest that a single GO surface display a semiconductor behavior^76–78^, in agreement with the literature reports employing a similar structure^41^. However, a higher band gap energy for the GO structure with higher oxidation states was observed, in contrast to literature reports^76^. The C:O ratio deals with the electronic behavior of the designed GO, in which a higher ratio yields a shorter band gap energy^79^. A higher conductivity achieved with GO is attributed to the presence of the sp^2^ carbon affecting the conductivity of the structure. Herein, the GO employed a C:O ratio of 9:1, attributed to six oxygen atoms from the functional groups with 56 carbon atoms as a majority decorated onto the surface. As a consequence, the structure attains a smaller band gap energy, resulting in the semiconductor character at a lower oxidation state^76,80^.

The higher band gap energies found in each isolated NIPAM and nucleobase suggests that each structure owns stability rather than being reactive. The observed band gap energies represent an insulating character, confirming that the presence of NIPAM molecule tend to induce greater stability into the GO structure, supported by a slight increase in the band gap energy once the NIPAM was assembled.

Regarding the nucleobase in the GO complex, the energy gap decreases upon adsorption of four nucleobases. For instance, after adsorption of A and G on the GO surface, the band gap energy of GO changed from 0.631 eV to 0.610 eV and 0.577 eV, respectively. The variation in the band gap energies is attributed to the different elements on the nucleobases, with an increasing trend from G, A, T, to C. The presence of nitrogen and an additional five-membered ring of A and G nucleobases increases the probability of sharing a higher level of electronic interaction towards the GO^71^. Interestingly, this trend is analogous with the adsorption energy trend of nucleobases in GO complexes. A similar approach has been described in a previous study employing a graphite surface^71,81^. These conductivity properties correlate exponentially toward the negative value of the energy gap. Hence the adsorption of nucleobases on GO surface enhances a significant change for the electronic conductivity in this case.

However, the trend for the band gap energies of A < G < C < T observed for the nucleobases on the GO/NIPAM complex is contradictory to its adsorption energies. This is attributed to the presence of NIPAM molecule which may lead to a narrower band gap energy and together with its insulating properties, resulting in the GO molecule to be rather non-reactive, thus affecting the conductivity in adsorbed nucleobases. As a consequence, the profile of the nucleobase molecule on the GO/NIPAM complex tends to be inconsistent towards the adsorption energy. This phenomenon may also be strengthened due to the impact of the imperfect quantitative description of the GGA, which immediately underestimates the energy in the complex structure^82,83^.

The global reactivity of the isolated molecule as well as the adsorbed nucleobases in the GO and GO/NIPAM complexes are presented in Table S8. The results illustrate the global hardness (η) and chemical potential (µ) of each molecule before and upon adsorption to both surfaces. Approaching the NIPAM molecule on the GO surface increases the η value of GO from 0.316 eV to 0.328 eV, which is a coincidence with its increasing trend of the band gap energies, but in contrast to the µ value which shows an increase from −4.229 eV to −4.116 eV. The greater η value reflects greater stability or lower reactivity of the system. On the other hand, the variation of µ does not follow any regular trend since it describes the escaping potency of electrons from the molecular system in an equilibrium state and in turn, a higher µ value suggests more reactivity and less stability of the species^52,84^.

Additionally, the adsorption of the nucleobases onto the GO or GO/NIPAM surface decreases the η at four studied nucleobases complexes (Table S8). For instance, with approaching G molecule to the GO and GO/NIPAM surfaces, the η values turn from 0.316 eV into 0.288 eV and from 0.328 eV into 0.314 eV, respectively. This suggests that adsorption of nucleobase molecule in GO or GO/NIPAM surface enables to enhance the reactivity of the complex. The η values vary due to the different HOMO and LUMO characterised by each nucleobase structure toward each surface. Therefore, their values gain a similar trend with the electronic band gap energy. Further, the overall η values of the nucleobases in GO/NIPAM are higher than those in GO, in accordance with the band gap energies observed for the adsorption. Due to the presence of NIPAM in GO, the GO/NIPAM complex generates quite a lower reactivity as compared to the nucleobases in the GO surface, indicative by the higher band gap energies. Moreover, the µ values of the nucleobase complexes in GO and GO/NIPAM increases upon adsorption. However, even though this reactivity of the adsorbed nucleobases increase towards the GO and GO/NIPAM, the entire system at both surfaces are thermodynamically stable^84^. In correlation with the insulating property of NIPAM, these results confirm that the functionalisation of NIPAM tends to render the stability to GO rather than to their reactivity.

**Figure 2:**
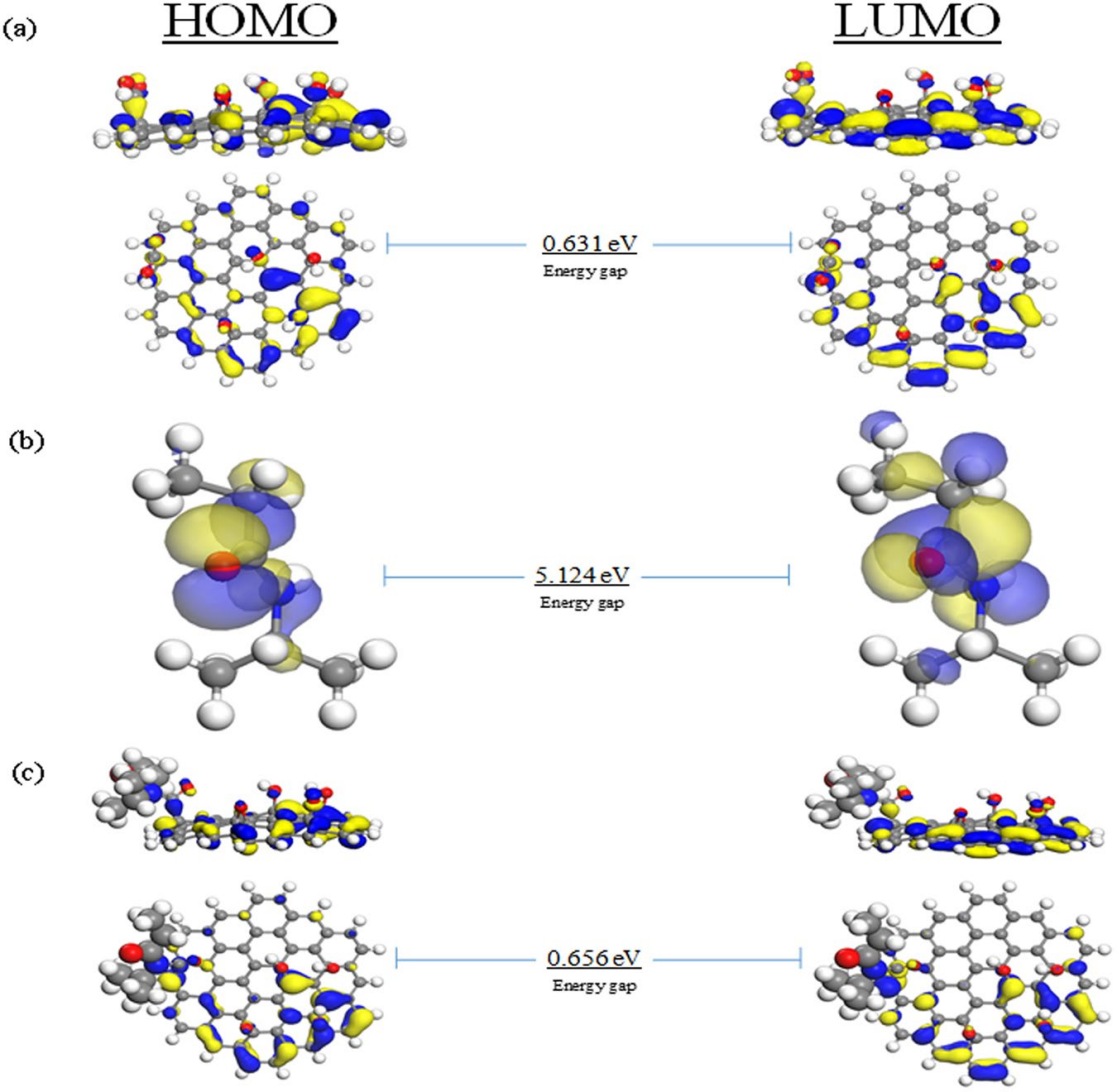
The isosurfaces of HOMO and LUMO of (**a**) GO, (**b**) PNIPAM monomer, and (**c**) GO/NIPAM calculated by GGA/DNP.

The frontier molecular orbital behavior of the individual molecules and their complexes can be defined qualitatively through the electronic distribution models. The HOMO-LUMO orbitals of the GO, NIPAM, and GO/NIPAM are shown in Fig. 2. For the GO surface, the electron moiety of the HOMO spreads evenly, typically on the sp^2^ and sp^3^ carbon conjunctions, while the LUMO isosurface is mostly delocalised around the oxygen functional groups (Fig. 2(a)). In the case of the single NIPAM molecule, the HOMO and LUMO isosurfaces are uniformly present along its backbone, particularly in the oxygen and nitrogen atoms (Fig. 2(b)). However, the modification of GO with NIPAM molecule shows no significant changes in the locations of GO orbitals isosurfaces (Fig. 2(c)). These results suggest that the entire surface of GO generates a polarised electronic transfer. Interestingly, both HOMO and LUMO moieties are identified and relied on the atoms of the carboxylic groups, where the NIPAM is attached. This confirms that there is possibly an electronic transfer from the GO surface through carboxylic group to the NIPAM.

Further, the electronic distributions of the nucleobases adsorption in GO and GO/NIPAM complexes have been examined as follows. The HOMO (Fig. S5) and LUMO (Fig. S6) isosurfaces in each complex of the nucleobase and GO are distributed on the GO surface in which, they remain the same with the isolated GO surface. There is no significant hybridisation evidenced between the nucleobase and the surface. Upon closer inspection, most nucleobases are not significantly involved in the electronic contribution of both orbitals. A slight variation is indeed recognised on the GO surface of the G system with fewer HOMO contours delocalised as compared to other nucleobases. Another minor isosurface of the HOMO is also observed closer to the nitrogen of C molecule in its complex (Fig. S6). The fewer HOMO regions may indicate that secondary electrons are localised in the G complex rather than in other nucleobases. Whereas, HOMO isosurface present closer to the C molecule probably indicates more electrons are condensed within the C and GO complex as compared to its LUMO level. Fewer isosurfaces in the HOMO may suggest that the particular complex gains fewer electrons in its HOMO, in turn, and the complex is less stable but more reactive to transfer electrons to the next level of orbitals (LUMO). Conversely, the higher HOMO isosurfaces may be associated with the more stable complex and less reactive in the transference of the electrons. These observations correlate with the band gap energy trends of GO complex for the G complex as the lowest and C complex as the widest energy gaps.

A similar trend is observed for every nucleobase at the GO/NIPAM complex, where most orbitals lie on the surface area (Figs S7 and S8). The HOMO isosurface is localised evenly along the edges of the GO and mostly delocalised on the oxygen functional groups area of GO surface (Fig. S7). The LUMO region, on the other hand, is delocalised on the similar area near the HOMO isosurface (Fig. S8). No visible alterations are observed in the carboxylic atom group after nucleobase adsorption, especially at the C12, C58, and oxygen atoms of the GO/NIPAM surface. Finally, it should be emphasised that the nucleobases adsorption on GO or the GO/NIPAM complex typically characterises physical adsorption trends, as evidenced by the absence of the electronic level perturbation upon the adsorption process, and the contribution of the electronic conductivity triggered by the GO surface^50^.

The electrostatic potentials (ESPs) of complexes are illustrated in Figs S9 and S10. In general, the ESPs are used to determine the properties of the intermolecular system. The calculated ESP strongly relies on the models considered^85^. Fig. S9 shows the ESP isosurfaces with isovalue of 0.016 plotted on the nucleobase and GO complex. The yellow and blue coloured-areas refer to the negative electrophilic and positive nucleophilic charges, respectively.

As seen in Figs S9 and S10, each complex of the nucleobase in GO or GO/NIPAM yield more electrons mostly from the oxygen functional groups of the GO due to their higher electronegativity as compared to the carbon atoms. In some cases, for the nucleobases with the nitrogen atom on the corner angle, (A, G, and C nucleobases) has accumulated negative charges resulting from an electronic transfer. Notably, the complexation of nucleobases with the GO surface expands the negative lobes into the side of the GO surface opposite to the adsorption area^85^, similar to the GO/NIPAM complex. As illustrated in Fig. S10 the NIPAM functionalisation on the GO surface brings the negative charges only from its oxygen atom. Due to the ratio of oxygen and carbon groups, the distribution of the nucleophilic region is highly centred in its surface functional groups^86,87^. The resulting isosurface recognised between the two molecules indicates a charge transfer from the GO surface to the nucleobase.

The total density of states (TDOS) of the isolated GO, NIPAM, GO/NIPAM, and the nucleobases complexed with GO and GO/NIPAM revealed a better understanding of the interaction of the nucleobase with both surfaces. The NIPAM peaks showed an overlap towards the Fermi level, suggesting a wide-space electron transfer from the highest occupied to the lowest unoccupied states of the corresponding molecule (Fig. S11(a)). The discrete peaks and trends of the extensive band gap energy also suggest that the NIPAM is a type of non-conductive molecule.

Meanwhile, the TDOS of the GO molecule shows continued and scattered peak-states along the energy axis (Fig. S11(b)). The computed results of the TDOS for the modified GO with NIPAM are depicted in Fig. S11(c). It is evident that TDOS exhibits new peaks, especially around −22 eV, indicating the presence of the NIPAM molecule. Moreover, the peaks around the Fermi level remains the same, indicating that the attachment of the NIPAM molecule does not contribute significantly to the GO conductivity. These results are in accordance with the surface characteristics results obtained from the previous experimental reports using cyclic voltammetry (CV)^40^. By incorporation of nanoparticles, the integrated PNIPAM can exhibit the electro-active surface mechanism to GO.

The TDOS of the adsorbed nucleobase in the GO and GO/NIPAM complexes are calculated to elucidate the electronic properties upon the adsorption process. In comparison with the isolated nucleobase before adsorption, the peaks around the Fermi level in GO and GO/NIPAM relatively moved to negative energy, resulting in the narrowing of the band gap energy (Fig. S12). On the other hand, the behavior of the negative peaks in GO and GO/NIPAM complexes seem to shift towards a more negative value, suggesting that the adsorption on both surfaces change the nucleobase to be more conductive. Also, the presence of the NIPAM in GO surface influences the electronic states of a nucleobase to be less conductive as opposed to those in GO complex only, shown by less shifting peaks toward the negative axis. For instance, the last peak of G in the GO system (Fig. S12) present at more negative axis compared to G at GO/NIPAM system and G as the isolated molecule. Similar patterns are also observed for the remaining three adsorbed nucleobases. These phenomena have been confirmed by the band gap energy results, due to its poor conductivity characteristic, NIPAM molecule tends to increase the band gap energy of the GO and complex surfaces of nucleobase adsorptions, suggesting a lower electronic transition as compared to the functionalised GO system.

### Molecular dynamics

The aforementioned DFT calculations were carried out to elucidate the details of the molecular interaction of the sensing applications on the GO surface through electronic properties calculation, with NIPAM-grafted GO surface complexed with the single nucleobase. It characterised the interaction behavior of the nucleobases on the surface of GO in terms of the binding strength and electroconductivity with or without the presence of an insulating monomer (NIPAM). Further, to evaluate the unique conformational changes of the coil and globular states of PNIPAM and its role in triggering the interaction between the aptamer and protein under typical critical temperature, the MD simulations of the PNIPAM/GO were carried out along with the binding simulation of protein with the aptamer.

The PNIPAM typically transform its configurational state into a coil or globular condition depending upon the temperature around its lower critical solution temperature (LCST) of 32 °C (~305 K). While the DFT is a quantum mechanical method to investigate single atom within the molecular stage from the electronic behavior point of view, the MD simulations is an exponential scaling of the quantum mechanical method itself based on classical force field which explains the connection of atom-to-atom in the more complex molecular system through nuclear coordinates calculation^88^. Therefore, to confirm the reliability of the bigger molecules of PNIPAM in response to its stimuli, the single chain of PNIPAM was firstly simulated under two different temperatures at 298 K and 310.7 K. Fig. S13 shows the radius of gyration, R_g_, over the production simulation time of 10 ns and 15 ns for 298 K and 310.7 K, respectively. The R_g_ defines the compactness of the specific conformation size of the polymer, that can be calculated by correlating the mass (*m*_*i*_) of the atom (*i*) within polymer with internal coordinate (*r*_*i*_) of the atom (*i*) of conformation:

$${R}_{g}={(\frac{{\sum }_{j}{\Vert r\Vert }^{2}{m}_{i}}{{\sum }_{i}{m}_{i}})}^{\frac{1}{2}}$$

The molecular interactions between PNIPAM and the GO surface are determined as a function of temperature. As shown in Fig. S13, the R_g_ at 298 K is higher compared to 310.7 K with a simulation time of 10 ns, indicating that the formation of polymer is more compact at above LCST (red line) rather than at 298 K. The R_g_ at both temperatures showed similar length in the initial time. After 1500 ps, the R_g_ at 310.7 K started to decrease the linearity to about 9 Å, while it showed flexible and more extended at 298 K (~23 Å). Indeed, the PNIPAM molecule consists of repeating units of hydrophobic and hydrophilic groups which allows it to undergo a transition into water-soluble or insoluble order depending on the temperature thus, resulting in the conformational change. It exists in the water solution as a coil formation, due to the domination of enthalpy contribution from the hydrogen bonding between water and the amide groups below LCST. When the temperature is greater than the LCST, its hydrogen bonds are weakened since the kinetic energy is greater than the energy of hydrogen bonding. As a consequence, the solvation entropy of the hydrophobic interaction between the isopropyl groups and its backbone is dominant and induces more intra/intermolecular aggregation of the hydrophobic groups, altering the polymer conformation from the flexible coil to a globular state^89–92^. This behavior can be supported by Fig. S14 as a result of different temperature applications. At the initial step, the PNIPAM chain is linear and is positioned at the middle (Fig. S14(a)). After 10 ns, the chain is linear and moved to the edge of PBC box at 298 K due to the effect of the dynamics (Fig. S14(b)), while it is found in a globular state at the end of simulation at 310.7 K (Fig. S15(c)).

In the next step, the construction of the similar structure of the PNIPAM system involving GO, aptamer and the protein molecules was performed. Furthermore, the simulations are categorised into two systems. The first system (System I (GO/PNs/Apt)) was built to attain the conformational change of the PNIPAM-grafted GO at different temperatures below and above LCST at 298 K and 310.7 K. Once the conformational transition was obtained, the protein molecule was introduced into the system as the second stage (System II (GO/PNs/Apt/pro)), to interact with the aptamer under the same temperature as in the first stage. Due to the constraints of a large number of atoms and computationally expensive, the time duration of the system I was set to 5,000 ps in Fig. S15, the kinetic energy of the system is quite stable with fewer fluctuations observed during the given time and under different temperatures. The increasing temperature breaks the hydrogen bonds and increases the kinetic motions of the molecules in the system; therefore, the kinetic energy of the system at 310.7 K is higher than that of 298 K.

**Figure 3:**
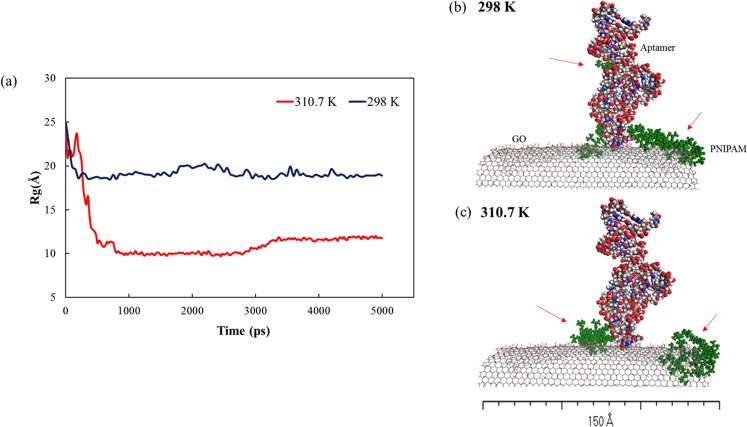
(**a**) Radius of gyration of the grafted PNIPAM on the System I at temperatures of 298 K (blue line) and 310.7 K (red line). The MD snapshots of the grafted PNIPAM in System I (green molecules) with behavior checked after MD simulations at (**b**) 298 K and (**c**) 310.7 K. These explain the natural properties of PNIPAM which are coiled under LCST and collapsed above LCST.

Figure 3(a) shows the R_g_ of the grafted PNIPAM chains after 5 ns of simulation at 298 K and 310.7 K. The R_g_ indicates the alteration of chains distance in average, over 5 ns intervals taken from 298 K and 310.7 K dynamics simulations. In general, the values of the R_g_ at 310.7 K is smaller than the corresponding value at 298 K. The R_g_ values of 310.7 K (red) are around 11 Å which are smaller than that at 298 K (blue) at 19 Å. As depicted in (Fig. 3(b)), the grafted chains (green molecule) are more extended at 298 K and observed to be collapsed in the globular form at 310.7 K (Fig. 3(c)). These findings are consistent with the previous experimental studies of PNIPAM functionalised-GO surface^93,94^. The assembly of PNIPAM on the GO substrate enabled to induce a coil-state under LCST (298 K) as well as globular-state at above LCST (310.7 K).

In System II, the protein is involved in the simulation with a Wy5a aptamer. The protein was hovering over the grafted surface nearby the binding region of the aptamer at approximately 5 Å. The initial orientation was approximated using the best-score pose predicted in the docking study according to the computational result, its adequate parallel sampling allowed to determine a molecular insight into adsorption. Within these simulations, the GO, PNIPAMs, and aptamer atoms were constrained while other elements including solvent were free to move. Herein, the MD simulations were only performed in 500 ps due to the computational cost implications under the progress of the cluster.

**Figure 4:**
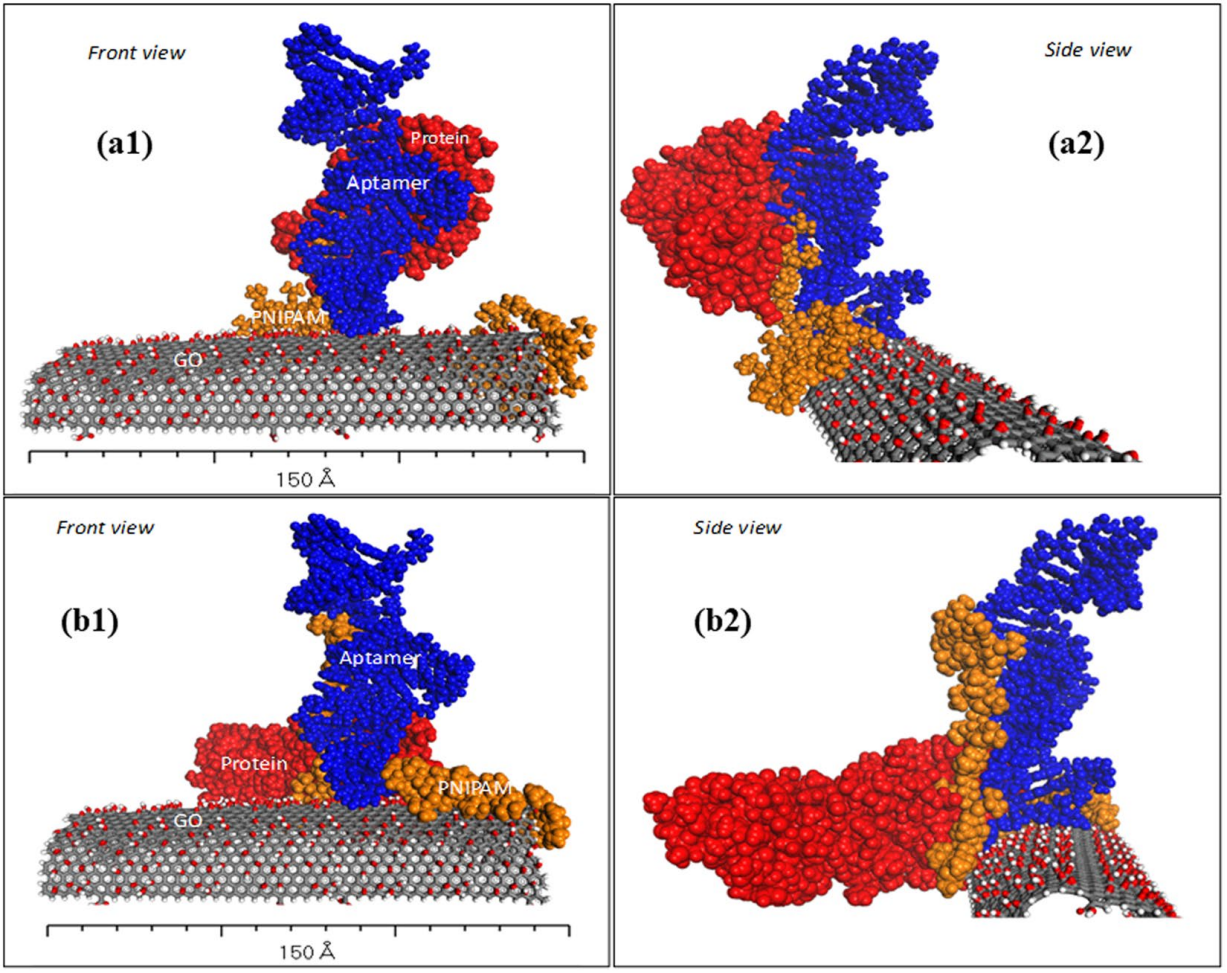
The depiction of the end-simulation of interaction studies between Wy5a aptamer and α6β4 protein at 310.7 K.

The interaction of the protein with a Wy5a aptamer is stronger at 310.7 K than 298 K as presented in Fig. 4(a1–2,b1–2). The extended chains of PNIPAM at 298 K seems to “turn-off” the protein interaction with the aptamer, thus preventing any interactions. This explains why the protein is hindered by the PNIPAM chain to reach the aptamer when the system was at 298 K, and due to the hydrophilic disturbance of PNIPAM, it seems to be shifted from its initial orientation. In contrast, when the temperature increases to above LCST, the protein is exposed (“turn-on” mode) and is subsequently able to bind to the aptamer. The access to the interaction region at 310.7 K is open, thus allowing the protein to bind with the aptamer. These are further confirmed by the distance calculations within the protein and the aptamer, in which the lower distance indicates a closer distance at a temperature of 310.7 K than 298 K (Fig. S16).

For the system in each temperature, the interaction energy between the surface complex and the protein was calculated using a single point energy calculation to yield the sufficient binding energy of protein on the interactive surface. The interaction energies were lower at 310.7 K compared to 298 K, suggesting a stronger interaction between the protein and aptamer (Table S9).

**Figure 5:**
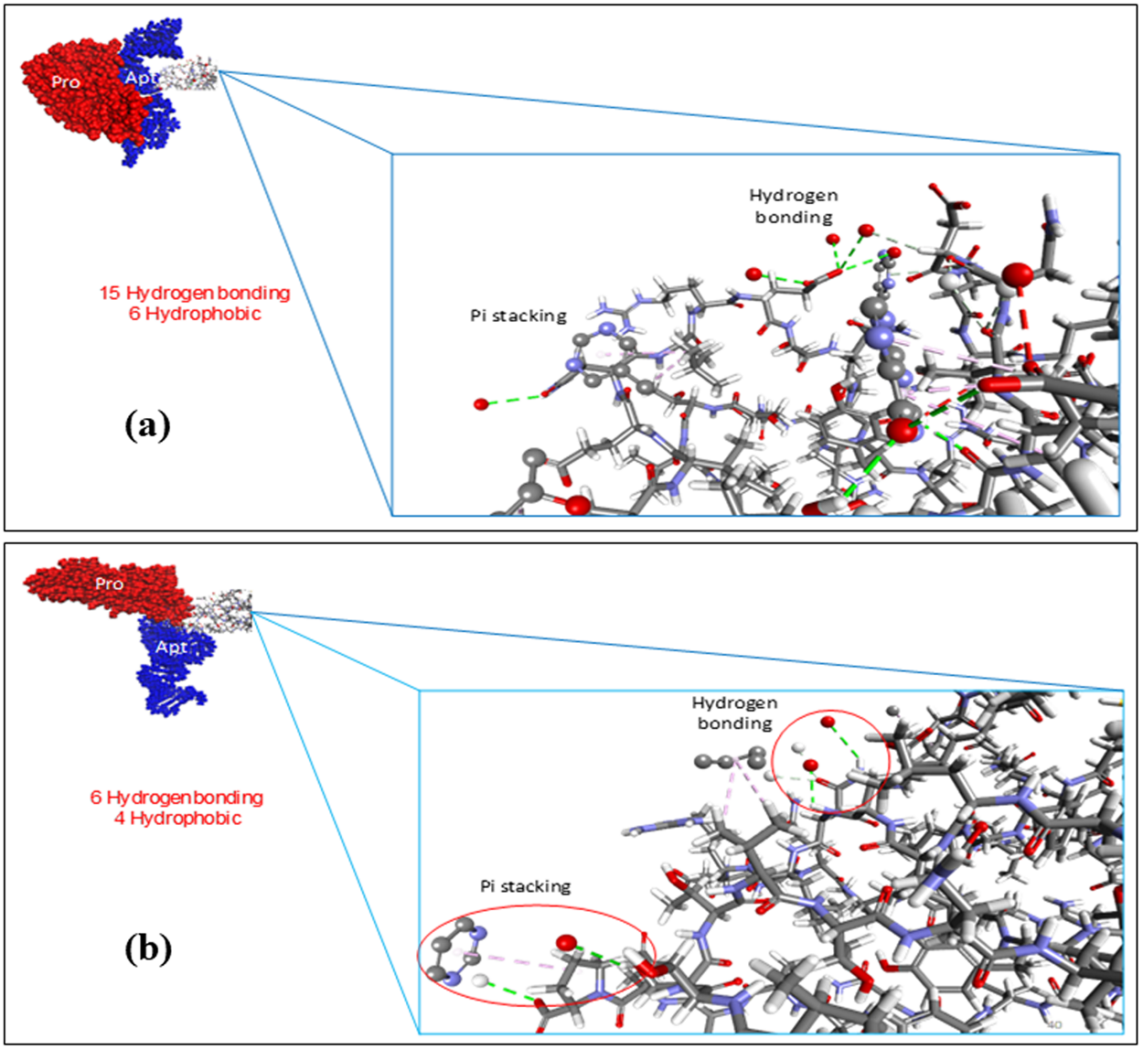
The interaction of the protein-aptamer in: (**a**) 310.7 K involving 15 hydrogen bonds and 6 hydrophobic interaction; and (**b**) 298 K involving 6 hydrogen bonds and 4 hydrophobic interaction.

Since the non-covalent interaction usually takes part in affecting the binding affinity, the number of binding interactions as well as the most binding types involved within the molecules were thereby investigated. The different molecular interactions are shown in Fig. 5(a,b), where a higher number of bond interactions has evidence at 310.7 K than at 298 K, that attributes to the non-covalent interactions, such as electrostatic, van der Waals, or hydrophobic. The van der Waals energy is calculated with a standard 12-6 Lennard-Jones potential while the electrostatic energy is with a Coulombic potential^63^. Computationally, these energy values were calculated using CHARMM forcefield incorporated in the Discovery Studio software upon the successful completion of dynamics simulation using COMPASS forcefield. The contribution of the binding is further analysed to be electrostatic and hydrophobic. There exist about 15 hydrogen bonds and six hydrophobic contacts which may exhibit stronger interaction at 310.7 K as compared to the system at 298 K with only six hydrogen bonds and four hydrophobic contacts. It can be understood due to the more accessible binding region between the protein and aptamer stimulated by the tunable surface of PNIPAM when it is on the globular state.

## Conclusions

Herein, a theoretical principle was employed to establish the electrical nature of PNIPAM along with other surface components fabricated onto GO nanomaterials. DFT calculations elucidated the details of the molecular interactions of the GO surface through electronic property calculations, in which the NIPAM-grafted GO surface was complexed with a single nucleobase. The presence of the NIPAM monomer stabilises the system due to the interaction between the nucleobases and GO, confirmed by a higher band gap energy observed in the nucleobase and GO/NIPAM complex rather than with the nucleobase and GO complex. The interaction of nucleobases towards the surface (GO or GO/NIPAM) is mostly stabilised by hydrogen bonding. This is also evident with an incremental trend observed in adsorption energy between GO/NIPAM and nucleobases compared to the GO and nucleobases. These results further support the fact that the presence of PNIPAM provides a tunable surface between the aptamer and the protein, without influencing the electroconductivity of the central system. On the other hand, the conductivity role is greatly enhanced by the presence of the GO surface.

Finally, the thermal-responsive behavior of the PNIPAM-grafted GO surface has successfully been simulated through MD simulations and positively induces an “on”/“off” surface-state as the temperature changes around its LCST. It is worth mentioning that this behavior contributed to the interaction between the Wy5a aptamer and α6β4 protein, resulting in a stronger interaction observed at 310.7 K rather than 298 K. However, the strategy to integrate computational and experimental methods, would greatly benefit the advancement of biosensing platforms for medical applications.

## Supplementary information

Supplimentary information
