# Supplementary material for: Computational studies on the molecular insights of aptamer induced poly(N-isopropylacrylamide)-graft-graphene oxide for on/off- switchable whole-cell cancer diagnostics
Source: Sci Rep. 2019 May 27;9:7873. doi: 10.1038/s41598-019-44378-x (PMC6536548; doi:10.1038/s41598-019-44378-x)
Supplement: Supplementary file 1 — Supplimentary information [file 41598_2019_44378_MOESM1_ESM.docx]

Supplementary Information

**Computational studies on the molecular insights of aptamer induced poly(*N*-isopropylacrylamide)-*graft*-graphene oxide for on/off- switchable whole-cell cancer diagnostics**

**Athika Darumas Putri^1,2^, Bayu Tri Murti^1,2^, Suvardhan Kanchi^1^, Myalowenkosi I. Sabela^1^, Krishna Bisetty^1*^, Ashutosh Tiwari^3,4*^, Inamuddin^5,6*^, Abdullah M. Asiri^5,6^**

# **The Docking Method**

## ***Preparation of Aptamer Structure***

The Wy5a aptamer was selected based on the best docking results among the other aptamers candidate. To obtain prediction of the secondary structure (2D) of aptamer folding with more information details, Mathews Lab RNA Structure software was used (available online at http://rna.urmc.rochester.edu/RNAstructure.html). To achieve a three-dimensional aptamer structure, MFold software was utilized (Zuker and Jacobson, 1998, Lee et al., 2015) to fold the initial sequence of each aptamer into its secondary structure. The folding temperature was set to 25^0^C with solvent condition in default setting, the dot-bracket (Vienna) format was fetched for tertiary (3D) prediction. For this purpose, RNA Composer software was applied to generate the 3D structure automatically. The Vienna format imported to the RNA Composer was generated into PDB (protein data base) format of 3D structure as outcome. Notably, the RNA Composer only generates the Vienna format input into the PDB file of RNA. If compared to ssDNA aptamer structure, hydroxyl group appeared at 2’-carbon atom of ribose, while uracil was at the thymine position. The Discovery Studio Visualizer (or ver.2016) was employed to adjust this condition into ssDNA format (Hu et al., 2015, Heiat et al., 2016, Ahirwar et al., 2016).

## ***Preparation of Protein Structure***

The α6β4 protein structure was extracted into PDB file (code: 1QG3) from RSCB biomolecular bank. Before protein-protein docking, the water and ligand molecules were removed and hydrogen atoms were added in the protein structure. Each structure of aptamer and protein were adjusted first using Dreiding-like force field tool to pre-optimize the structural geometry. The minimization processes were done by using steepest descent minimization of 32,000 steps using CHARMm force field under Discovery Studio Ver. 2016. ZDOCK molecular docking and ZRANK refinement module as part of Discovery Studio ver.2016 were utilized to provide docking prediction and reranking refinement, respectively. ZDOCK was used to perform coarse-grained search of binding characteristics between the aptamers and α6β4 protein. According to this, ZDOCK-ZRANK has been widely employed for complex systems prediction between protein-protein and peptide-nucleic acid (Chen et al., 2003, Huang et al., 2015, Hu et al., 2015, Xia et al., 2016). The 6^o^ sampling was employed in ZDOCK to attain more accurate movement of 54,000 of the predictive complexes. The first top 2,000 ranks were subsequently carried out for the interaction analysis with ZRANK to achieve more accurately reranking the docked complexes.

**Table S1.**

The optimized configuration of PNIPAM monomer on the A-GO and A-GO/NIPAM systems before and after nucleobases adsorption calculated by GGA/DNP.

|  | Geometry Structures | Optimized Structures | |
| --- | --- | --- | --- |
|  |  | A-GO | A-GO-NIPAM |
| Bond length (Å) | C11-C12 | 1.494 | 1.491 |
|  | C12-C13 | 1.516 | 1.511 |
|  | C12-C58 | 1.630 | 1.636 |
|  | C12-C28 | 1.504 | 1.508 |
| Bridging angles (^0^) | C11-C12-C13 | 111.894 | 111.352 |
|  | C13-C12-C28 | 112.650 | 112.924 |
|  | C13-C12-C58 | 103.157 | 104.732 |
|  | C28-C12-C11 | 112.680 | 112.825 |
|  | C11-C12-C58 | 109.853 | 113.624 |
|  | C58-C12-C28 | 105.969 | 100.711 |

**Table S2.**

The optimized configuration of PNIPAM monomer on the C-GO and C-GO/NIPAM systems before and after nucleobases adsorption calculated by GGA/DNP.

|  | Geometry Structures | Optimized Structures | |
| --- | --- | --- | --- |
|  |  | C-GO | C-GO-NIPAM |
| Bond length (Å) | C11-C12 | 1.495 | 1.492 |
|  | C12-C13 | 1.516 | 1.509 |
|  | C12-C58 | 1.634 | 1.630 |
|  | C12-C28 | 1.503 | 1.508 |
| Bridging angles (^0^) | C11-C12-C13 | 111.978 | 111.900 |
|  | C13-C12-C28 | 112.619 | 112.702 |
|  | C13-C12-C58 | 103.124 | 105.382 |
|  | C28-C12-C11 | 112.939 | 113.304 |
|  | C11-C12-C58 | 109.608 | 110.891 |
|  | C58-C12-C28 | 105.881 | 101.911 |

**Table S3.**

The optimized configuration of PNIPAM monomer on the T-GO and T-GO/NIPAM systems before and after nucleobases adsorption calculated by GGA/DNP.

|  | Geometry Structures | Optimized Structures | |
| --- | --- | --- | --- |
|  |  | T-GO | T-GO-NIPAM |
| Bond length (Å) | C11-C12 | 1.493 | 1.495 |
|  | C12-C13 | 1.516 | 1.512 |
|  | C12-C58 | 1.631 | 1.631 |
|  | C12-C28 | 1.503 | 1.505 |
| Bridging angles (^0^) | C11-C12-C13 | 111.884 | 112.193 |
|  | C13-C12-C28 | 112.722 | 111.915 |
|  | C13-C12-C58 | 103.403 | 105.385 |
|  | C28-C12-C11 | 112.541 | 113.235 |
|  | C11-C12-C58 | 109.573 | 100.682 |
|  | C58-C12-C28 | 106.106 | 112.661 |

**Table S4.**

The optimized configuration of PNIPAM monomer on the G-GO and G-GO/NIPAM systems before and after nucleobases adsorption calculated by GGA/DNP.

|  | Geometry Structures | Optimized Structures | |
| --- | --- | --- | --- |
|  |  | G-GO | G-GO-NIPAM |
| Bond length (Å) | C11-C12 | 1.496 | 1.492 |
|  | C12-C13 | 1.516 | 1.510 |
|  | C12-C58 | 1.625 | 1.634 |
|  | C12-C28 | 1.503 | 1.509 |
| Bridging angles (^0^) | C11-C12-C13 | 111.466 | 111.805 |
|  | C13-C12-C28 | 112.866 | 112.651 |
|  | C13-C12-C58 | 102.700 | 104.745 |
|  | C28-C12-C11 | 112.598 | 113.001 |
|  | C11-C12-C58 | 109.890 | 112.789 |
|  | C58-C12-C28 | 106.715 | 101.108 |

**Table S5.**

The comparison of atomic hydrogen bonding distance (in (Å)) between the optimized (O) and initial (I) nucleobase and GO/NIPAM complexes calculated by GGA/DNP.

| System | Intramolecular bonding | | | Intermolecular bonding | | |
| --- | --- | --- | --- | --- | --- | --- |
|  | Atom | Distance (O) | Distance (I) | Atom | Distance (O) | Distance (I) |
| A-GO/NIPAM | H3-O2 | 2.032 | 2.393 | N-H1 | 1.775 | 3.226 |
|  |  |  |  | H2-O1 | 2.115 | 2.999 |
| C-GO/NIPAM | H2-O2 | 2.070 | 2.501 | N-H1 | 2.072 | 2.373 |
|  |  |  |  | H4-O4 | 2.093 | 3.008 |
|  |  |  |  | H3-O3 | 2.133 | 2.509 |
| T-GO/NIPAM | O2-H2 | 2.389 | 2.408 | O1-H1 | 1.755 | 4.021 |
|  |  |  |  | O1-H2 | 2.494 | 2.129 |
|  |  |  |  | H3-O2 | 1.785 | 2.337 |
| G-GO/NIPAM | O1-H2 | 2.029 | 2.545 | O2-H1 | 2.180 | 2.212 |

**Table S6.**

The adsorption energies (in kJ/mol) distribution of GO and GO/NIPAM complexes with nucleobases calculated by GGA/DNP.

| **System** | **Adsorption Energies (kJ/mol)** | |
| --- | --- | --- |
|  | **GO/NIPAM** | **GO** |
| A | -41.940 | -120.786 |
| T | -82.752 | -114.043 |
| G | -66.368 | -121.654 |
| C | -51.956 | -104.326 |

**Table S7.**

The calculated energy gaps (eV) for pure nucleobases, GO, GO/NIPAM, and their complexes with nucleobases.

| System | Pure Molecule | | | + GO | | | + GO/NIPAM | | |
| --- | --- | --- | --- | --- | --- | --- | --- | --- | --- |
|  | HOMO | LUMO | Energy Gap | HOMO | LUMO | Energy Gap | HOMO | LUMO | Energy Gap |
| GO | -4.697 | -4.065 | 0.631 | - | - | - | - | - | - |
| NIPAM | -5.393 | -0.269 | 5.124 | - | - | - | - | - | - |
| GO/NIPAM | -4.444 | -3.788 | 0.656 | - | - | - | - | - | - |
| A | -5.391 | -1.505 | 3.886 | -4.452 | -3.842 | 0.610 | -4.357 | -3.739 | 0.618 |
| T | -5.957 | -2.136 | 3.820 | -4.408 | -3.793 | 0.615 | -4.503 | -3.859 | 0.645 |
| G | -5.059 | -1.108 | 3.951 | -4.452 | -3.875 | 0.577 | -4.408 | -3.780 | 0.629 |
| C | -5.548 | -1.859 | 3.690 | -4.585 | -3.957 | 0.629 | -4.498 | -3.861 | 0.637 |

**Table S8.**

Calculated global reactivity (eV) for pure nucleobases, pristine GO, GO/NIPAM, and their complexes with nucleobases.

| System | Pure Molecule | | + GO | | +GO/NIPAM | |
| --- | --- | --- | --- | --- | --- | --- |
|  | η (eV) | µ (eV) | η (eV) | µ (eV) | η (eV) | µ (eV) |
| GO | 0.316 | -4.229 | - | - | - | - |
| NIPAM | 2.559 | -2.796 | - | - | - | - |
| GO/NIPAM | 0.328 | -4.116 | - | - | - | - |
| A | 1.943 | -3.448 | 0.305 | -4.147 | 0.309 | -4.048 |
| T | 1.910 | -4.046 | 0.307 | -4.101 | 0.322 | -4.181 |
| G | 1.976 | -3.083 | 0.288 | -4.163 | 0.314 | -4.094 |
| C | 1.845 | -3.703 | 0.314 | -4.271 | 0.318 | -4.180 |

**Table S9.**

Interaction energy of the protein with aptamer under two different temperatures of 298 K (below LCST) and 310.7 K (above LCST).

| System | Total Energy (kcal/mol) | |
| --- | --- | --- |
|  | 298 K | 310.7 K |
| E_system_ | 162,142.17 | 161,213.84 |
| E_solvent_ | 39,788.83 | 40,230.59 |
| E_surface_ | 44,572.92 | 44,587.80 |
| E_protein_ | 2,491.59 | 2,226.53 |
| E_aptamer_ | 76,085.72 | 76,085.72 |
| E_interaction_ | -796.88 | -1,916.79 |

**Fig. S1.** The numbered atoms of the GO/NIPAM (a), GO (b), and NIPAM (c) molecules for DFT calculations.


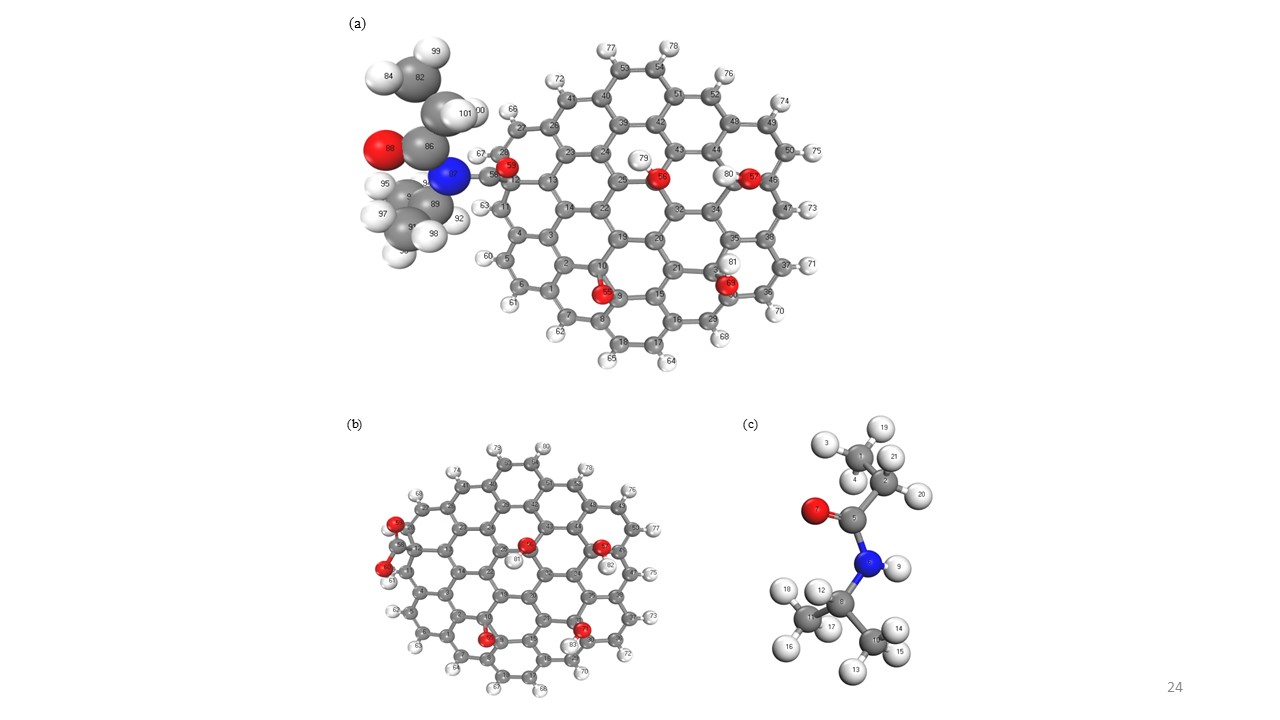

**Figure S2.** The optimized structures of nucleobases on GO-NIPAM complexes along with atomic hydrogen bonding orientation calculated by GGA/DNP.


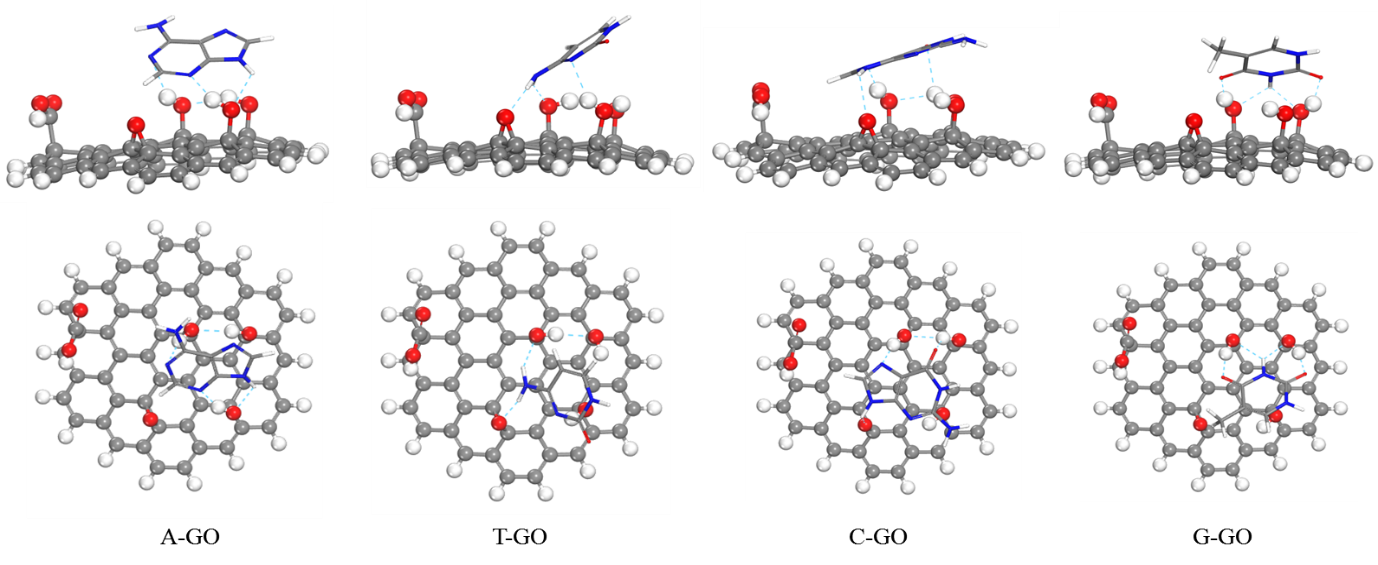


**Figure S3.** The optimized structures of nucleobases on GO complexes along with atomic hydrogen bonding orientation calculated by GGA/DNP.

**Figure S4.** Comparison of adsorption energies (in kJ/mol) of GO and GO/NIPAM complexes with nucleobases calculated by GGA/DNP

**Figure S5.** The HOMO isosurfaces of nucleobases and GO complex calculated by GGA/DNP.

**Figure S6.** The LUMO isosurfaces of nucleobases and GO complex calculated by GGA/DNP.

**Figure S7.** The HOMO isosurfaces of nucleobases and GO/NIPAM complex calculated by GGA/DNP.

**Figure S8.** The LUMO isosurfaces of nucleobases and GO/NIPAM complex calculated by GGA/DNP.

**Figure S9.** The isosurfaces of electrostatic potential of nucleobases and GO complexes calculated by GGA/DNP.

**Figure S10.** The isosurfaces of electrostatic potential of GO-NIPAM and nucleobases-GO/NIPAM complexes calculated by GGA/DNP.

**Figure S11.** The calculated total density of states of: (a) NIPAM, (b) GO, (c) GO/NIPAM molecules, and (d) the comparison among those in the scheme of DFT-D of GGA function.

**Figure S12.** Total of density of states of adsorbed nucleobase on GO and GO/NIPAM systems at DFT-D/GGA scheme.


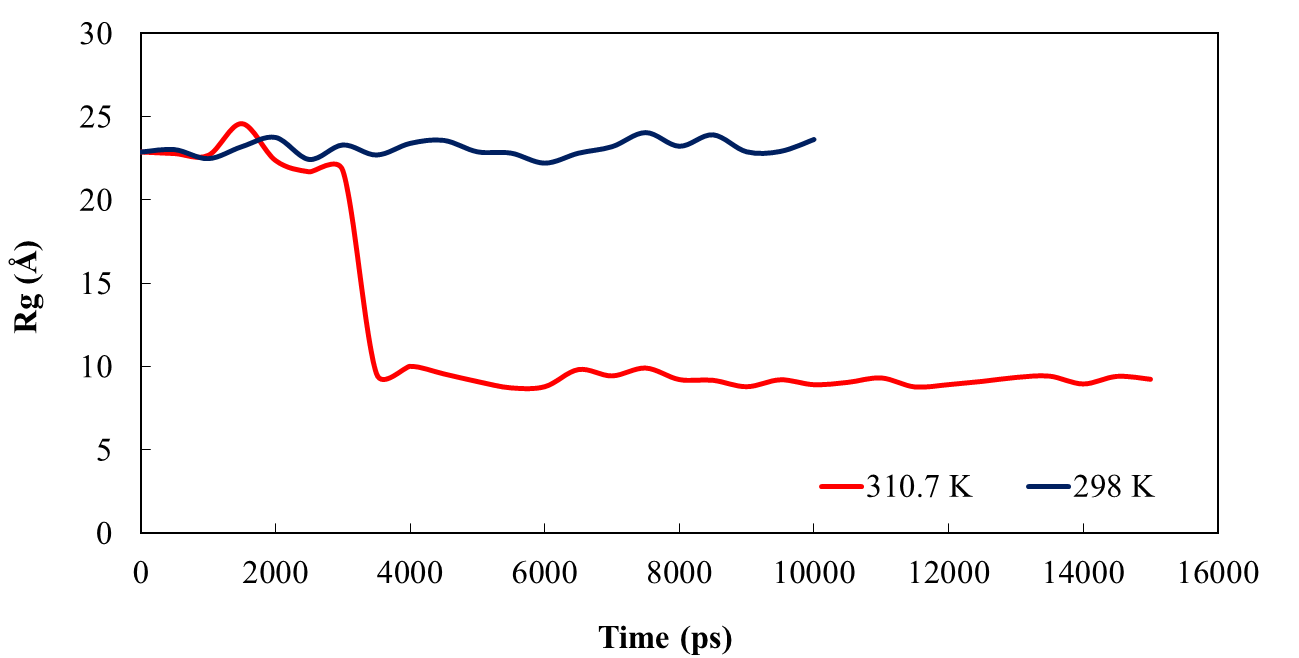


**Figure S13.** Radius of gyration of single PNIPAM at temperatures of 298 K (blue line) and 310.7 K (red line).


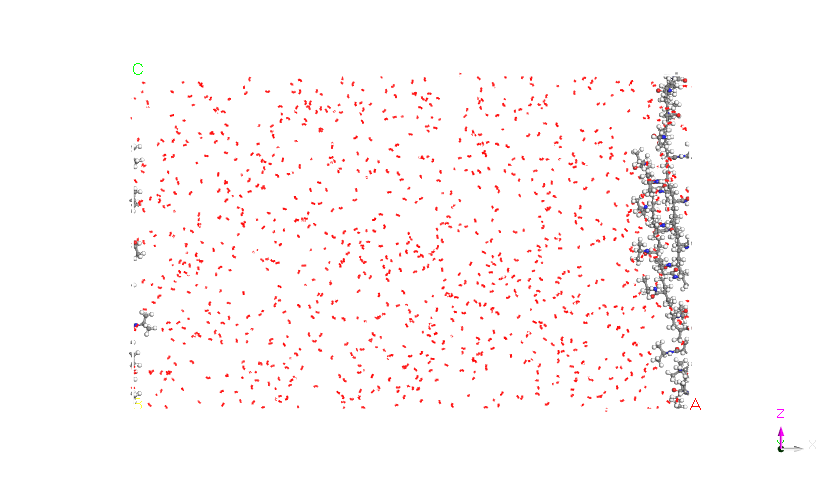

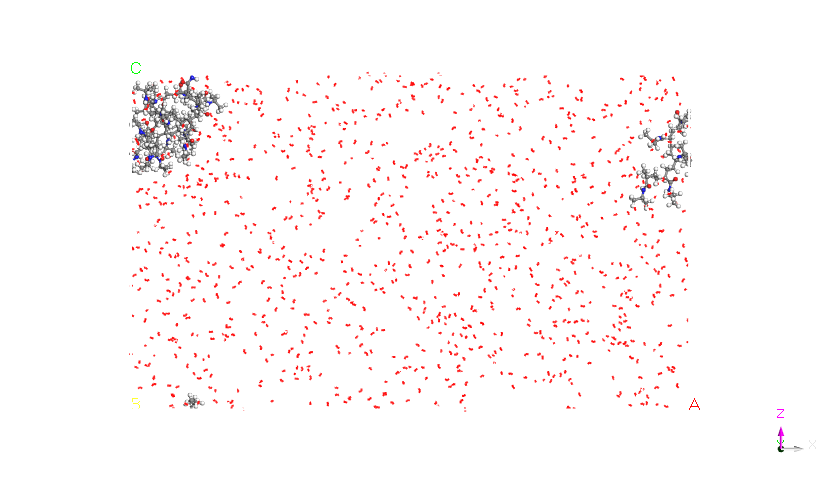

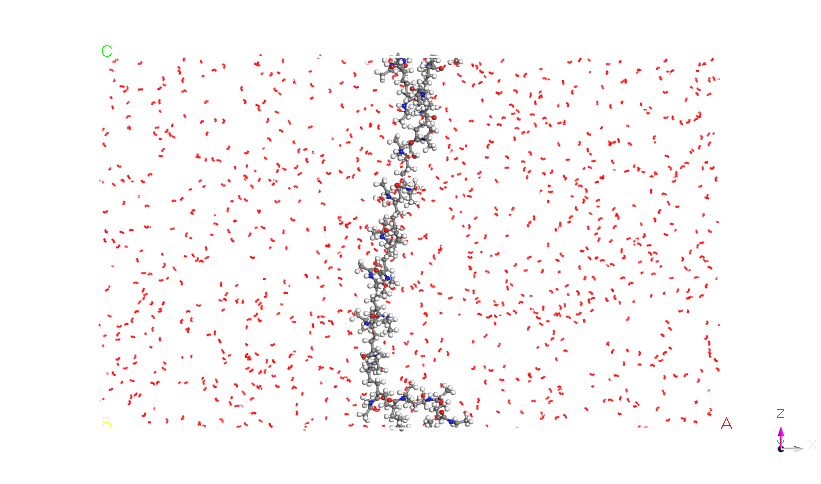


(a)

(b)

(c)

z

x

y

**Figure S14.** The MD snapshots of the single PNIPAM at the (a) starting simulation, and its behavior after MD simulations under (b) 298 K as well as (c) 310.7 K. These explain the natural properties of PNIPAM which is swollen above LCST and coiled under LCST temperatures.

**Figure 16.** (a) The kinetic energy and (b) temperature profiles of the System I at below (298 K) and above (310.7 K) LCST of molecular dynamics simulations.


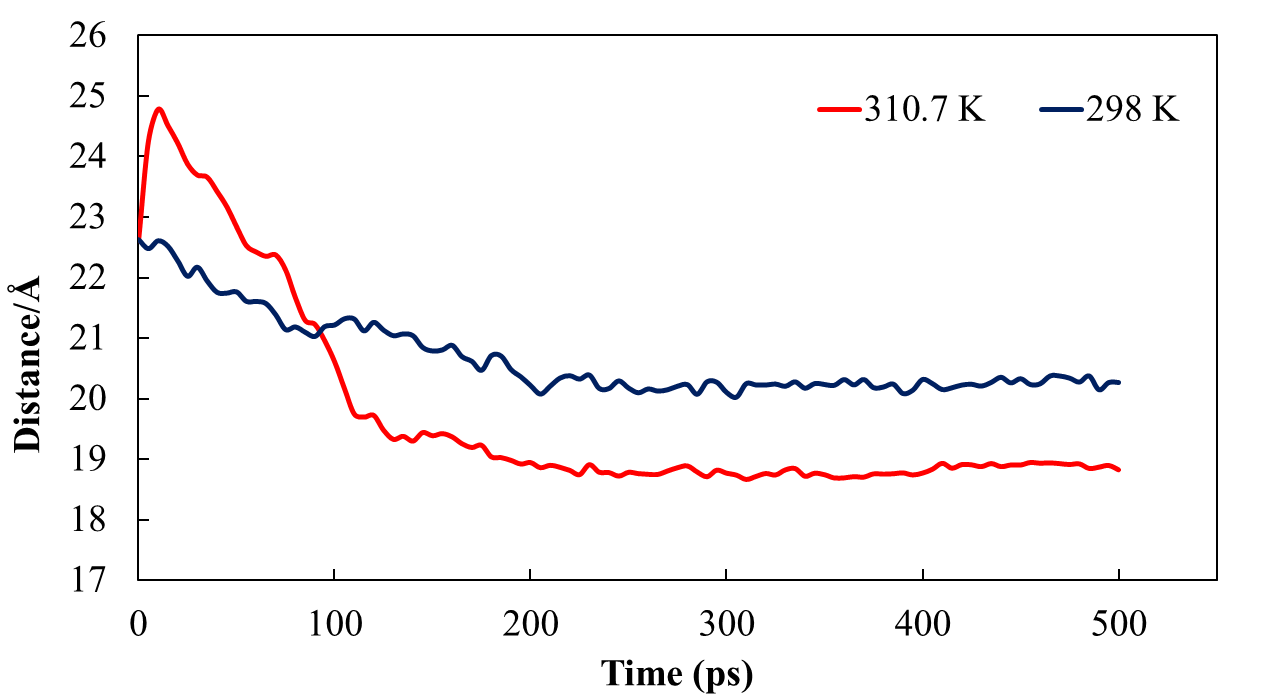


**Figure S16.** Distance between the protein and aptamer in the System II (GO-PNs-Apt-pro).
